# Clinical Characteristics and Risk Factors for Severe Immune-Related Liver Injury Following Nivolumab and Ipilimumab Combination Therapy

**DOI:** 10.3390/antib15050079

**Published:** 2026-09-01

**Authors:** Yuichi Yamazaki, Satoru Kakizaki, Yuki Tamura, Yumeo Tateyama, Tatsuma Murakami, Takuya Adachi, Hiroki Tojima, Toshio Uraoka

**Affiliations:** 1 Department of Gastroenterology and Hepatology, Gunma University Graduate School of Medicine, Maebashi 371-8511, Gunma, Japan; 2 Department of Clinical Research, NHO Takasaki General Medical Center, Takasaki 370-0829, Gunma, Japan

**Keywords:** immune checkpoint inhibitors, immune-related liver injury, nivolumab and ipilimumab, KL-6, cytomegalovirus

## Abstract

**Background**: Combination therapy with nivolumab and ipilimumab (Nivo-Ipi) has substantially improved outcomes in several advanced malignancies but is frequently associated with early-onset, severe immune-related liver injury (irAE-LI). This study aimed to identify pretreatment predictors of severe irAE-LI and to characterize its clinical course. **Methods:** We retrospectively analyzed 81 patients who received Nivo-Ipi combination therapy between 2018 and 2025. Severe irAE-LI was defined as liver injury requiring systemic corticosteroid therapy. **Results**: Severe irAE-LI developed in 11 patients (13.6%), with a median onset of 38 days after treatment initiation. More than half of the cases occurred after the first treatment cycle. This exploratory multivariable analysis identified baseline serum Krebs von den Lungen-6 (KL-6) levels of ≥400 U/mL as a potential biomarker associated with a strong preliminary risk signal for severe irAE-LI (odds ratio, 12.60; 95% confidence interval, 2.32–68.60; *p* = 0.003), whereas conventional liver function tests and inflammatory markers showed no significant association. The robustness of this signal was further confirmed by a sensitivity analysis excluding patients with lung adenocarcinoma (odds ratio 13.21, *p* = 0.005). Clinically, 54.5% of affected patients experienced steroid-refractory disease or relapse during corticosteroid tapering despite guideline-recommended high-dose corticosteroid therapy. Cytomegalovirus (CMV) reactivation was detected in 50.0% of patients tested on the basis of clinical indicators such as cytopenia or gastrointestinal symptoms, which may have contributed to a pseudo-refractory clinical course in some cases. **Conclusions**: Pretreatment KL-6 measurement may facilitate risk stratification before Nivo-Ipi therapy. In patients with steroid-refractory or relapsing severe irAE-LI, early introduction of mycophenolate mofetil together with CMV screening may improve clinical management.

## 1. Introduction

The advent of immune checkpoint inhibitors (ICIs), particularly the combination therapy with nivolumab and ipilimumab (Nivo-Ipi), has revolutionized the treatment landscape for various advanced malignancies [1,2]. By targeting programmed cell death-1 (PD-1) and cytotoxic T-lymphocyte–associated protein 4 (CTLA-4), this synergistic approach has demonstrated superior clinical efficacy compared with monotherapy [1,2]. However, this therapeutic success is accompanied by a significantly higher incidence of immune-related adverse events (irAEs). Immune-related liver injury (irAE-LI) is a major concern, occurring in up to 30% of patients receiving combination therapy [3,4,5].

Although high-dose corticosteroids remain the mainstay of treatment for severe irAE-LI [6], approximately 16–30% of cases exhibit steroid resistance or relapse, necessitating additional immunosuppressive agents such as mycophenolate mofetil (MMF) [7,8,9]. Furthermore, prolonged high-dose steroid treatment increases the risk of opportunistic infections, most notably cytomegalovirus (CMV) reactivation [10,11]. CMV infection can mimic the clinical features of irAEs, potentially leading to a “pseudo-refractory” course in which liver enzyme levels fail to improve despite intensified immunosuppression [10]. Although the association between CMV and steroid-refractory irAE colitis is well-documented, systematic data on CMV reactivation in the context of irAE-LI are scarce, and its impact on clinical management remains insufficiently characterized [11].

Identifying reliable pretreatment biomarkers for risk stratification of irAE-LI is a critical unmet need [5]. Various clinical factors have been explored, including female sex, prior ICI exposure, and baseline inflammatory markers such as the neutrophil-to-lymphocyte ratio (NLR) and C-reactive protein (CRP) [7,8]. More recently, the impact of host factors, such as metabolic syndrome and underlying liver status, has also gained attention [12]. However, the predictive accuracy of these factors remains inconsistent across cohorts, and no definitive pre-treatment biomarker for hepatotoxicity has been established in routine practice [13].

In current clinical practice, baseline patient evaluations prior to ICI administration often include a broad range of routine parameters, including markers used to screen for the risk of subclinical interstitial lung disease (ILD) [14,15]. In this study, we retrospectively analyzed 81 patients treated with Nivo-Ipi combination therapy to evaluate whether data from routine pretreatment screening could serve as novel predictors of severe irAE-LI.

## 2. Materials and Methods

### 2.1. Patients and Study Design

**Figure 1:**
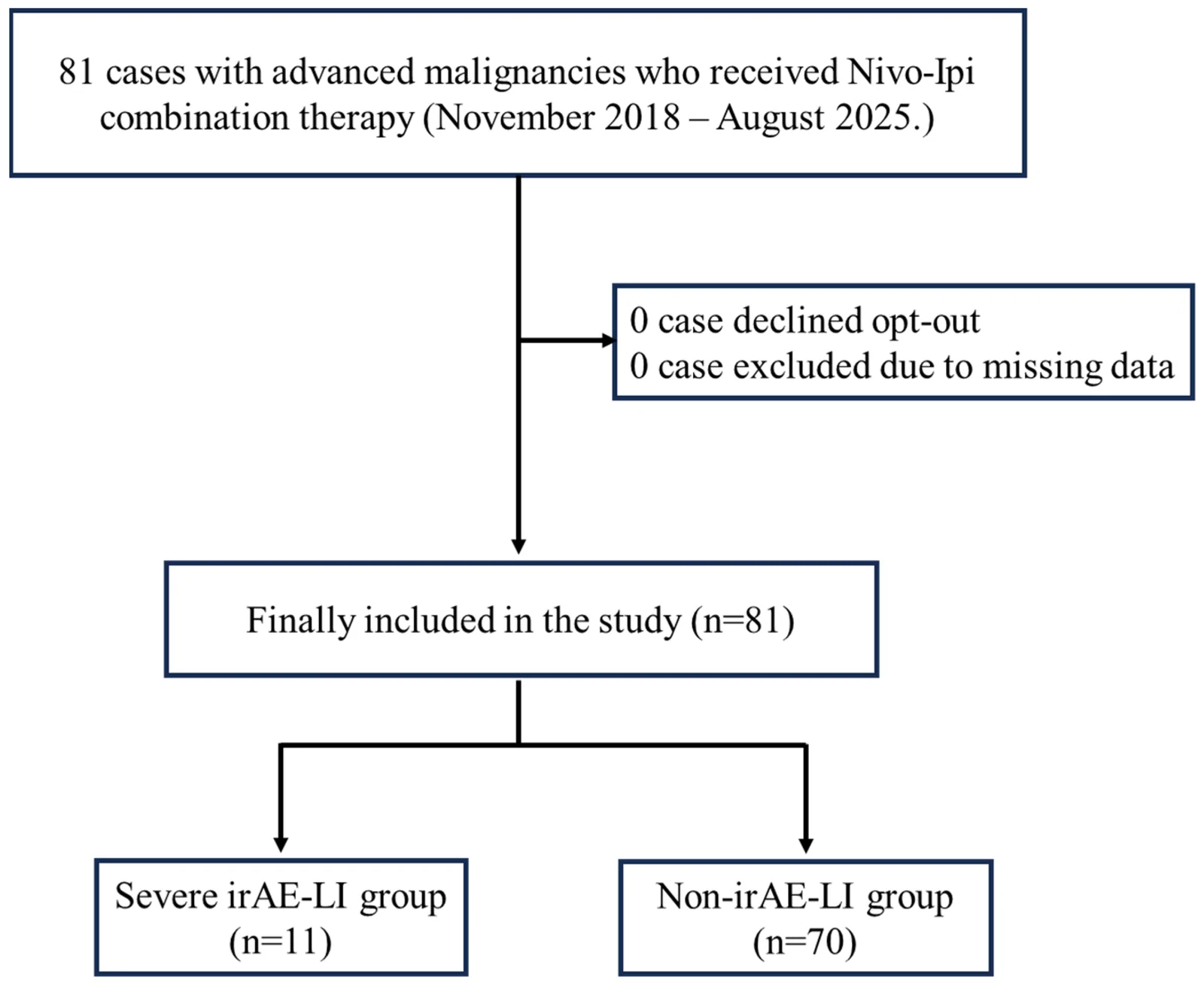
Patient flowchart of the study population.

This retrospective observational study was conducted at Gunma University Hospital. To minimize selection bias, we identified 81 consecutive patients who received combination therapy with Nivo-Ipi using a centralized drug administration database maintained by the Medical Information Management Department of Gunma University Hospital. Patients treated for various advanced malignancies–including malignant melanoma, renal cell carcinoma, esophageal cancer, lung adenocarcinoma, malignant pleural mesothelioma, and colorectal cancer–between November 2018 and August 2025 were screened. As illustrated in Figure 1 (Patient Flowchart), all 81 identified patients were included in the final analysis; no patients declined participation through the institutional opt-out mechanism, and no patients were excluded because of missing clinical or laboratory data. The study cohort was divided into two groups: the irAE-LI group (*n* = 11), consisting of patients who developed severe irAE-LI requiring corticosteroid therapy, and the non-irAE-LI group (*n* = 70). The study was conducted in accordance with the Declaration of Helsinki, and was approved by the institutional ethics committee of Gunma University (Approval number: HS2025-218; date of approval: 26 December 2025).

Flow diagram illustrating the selection and classification of patients treated with nivolumab and ipilimumab combination therapy between November 2018 and August 2025. A total of 81 consecutive patients were identified using a centralized drug administration database maintained by the Medical Information Management Department. No patients (0 cases) declined participation through the institutional opt-out consent mechanism, and no cases were excluded because of missing clinical or laboratory data. The final cohort of 81 patients was divided into two groups: the severe immune-related liver injury (irAE-LI) group (*n* = 11), comprising patients who required systemic corticosteroid therapy, and the non-irAE-LI group (*n* = 70). Source: Compiled by the authors.

### 2.2. Diagnosis and Differential Diagnosis

The diagnosis and classification of liver injury patterns, namely hepatocellular, cholestatic, or mixed patterns, were based on the R value in accordance with clinical practice guidelines for drug-induced liver injury (DILI) [16,17]. Severity was graded according to CTCAE version 5.0. To confirm the diagnosis of irAE-LI, we performed a rigorous differential diagnosis to exclude other etiologies, including viral infections. Screening was performed for hepatitis A virus, hepatitis B virus, hepatitis C virus (HCV), hepatitis E virus, Epstein–Barr virus, and CMV. Patients were also evaluated for autoimmune hepatitis (AIH), and computed tomography or magnetic resonance cholangiopancreatography (MRCP) was performed to rule out biliary tract diseases and progression of liver metastases. Although liver biopsy was considered an option in cases requiring differentiation from other liver diseases, it was not performed in any of the 11 patients in the irAE-LI group (0/11). This was because other etiologies were successfully excluded through clinical and laboratory evaluations, and because the rapid progression of severe liver injury necessitated the prompt initiation of corticosteroid therapy, with immediate clinical intervention prioritized over histological confirmation.

### 2.3. Detailed Clinical Evaluation of the irAE-LI Group (n = 11)

For patients in the irAE-LI group, we conducted a multidimensional review of the clinical course. Onset-related parameters included the time from the first ICI dose to the onset of irAE-LI and the total number of ICI doses administered before onset. Biochemical severity was assessed based on peak alanine aminotransferase (ALT) levels and the biochemical injury pattern at the time of corticosteroid initiation. Multisystem involvement was assessed by evaluating the presence and types of concurrent irAEs, including pneumonitis, colitis, and other organ-specific toxicities, based on clinical manifestations and laboratory findings.

### 2.4. Treatment Protocol and Steroid Refractoriness

In accordance with Japanese and Western guidelines [6,17,18], corticosteroids were administered for grade 3 or 4 liver injury and for worsening grade 2 cases. The standard initial corticosteroid regimen consisted of prednisolone (PSL; 0.5–1.0 mg/kg) or methylprednisolone (mPSL; 2.0 mg/kg). Until 2022, mPSL pulse therapy (1.0 g) was occasionally administered at the discretion of the attending physician as initial treatment for patients with grade 4 acute hepatitis-like onset. Since 2023, however, mPSL pulse therapy has no longer been used as an initial treatment strategy, in accordance with updated clinical guidelines.

CMV reactivation was diagnosed using an integrated clinical and microbiological approach. Microbiological positivity was defined as a positive result on the CMV pp65 antigenemia assay (≥1 positive cell/150,000 WBCs) or quantitative real-time PCR (CMV DNA ≥ 2.0 log_10_ copies/mL). These assays were performed by H.U. Frontier, Inc. (Tokyo, Japan). CMV testing was not performed systematically for all patients but was prompted by clinical indicators in patients receiving high-dose corticosteroids, such as a pseudo-refractory clinical course, unexplained cytopenia, or gastrointestinal symptoms.

Steroid refractoriness was defined as the absence of improvement in liver enzyme levels within 3–5 days after corticosteroid initiation. Relapse was defined as worsening of liver enzyme levels during corticosteroid tapering. Interventions for steroid-refractory disease or relapse included increasing the corticosteroid dose, adding MMF, or administering secondary mPSL pulse therapy. Until 2022, secondary mPSL pulse therapy was occasionally performed at the discretion of the attending physician for PSL-refractory cases or relapse during PSL tapering. Since 2023, secondary mPSL pulse therapy, not recommended by current guidelines, has no longer been performed.

For monitoring, regular CMV-DNA screening using polymerase chain reaction (PCR) was conducted in patients with prolonged, steroid-refractory, or relapsing courses to detect reactivation during high-dose immunosuppression.

### 2.5. Data Collection and Biological Markers

Baseline demographic data including body mass index (BMI) and comorbidities, including dyslipidemia, hypertension, diabetes mellitus, alcohol use, smoking history, and chronic liver disease, were collected. BMI was calculated as weight in kilograms divided by the square of height in meters (kg/m^2^). Laboratory data obtained before the first Nivo-Ipi dose included aspartate aminotransferase (AST), ALT, alkaline phosphatase (ALP), gamma-glutamyltransferase (γ-GTP), total bilirubin (T-Bil), albumin (Alb), prothrombin time–international normalized ratio (PT-INR), and CRP. We also comprehensively evaluated the following biological markers before the first Nivo-Ipi dose: the lung-related marker Krebs von den Lungen-6 (KL-6); inflammatory and immune markers, including the NLR and antinuclear antibody (ANA) titer, with ANA positivity defined as a titer of ≥40; and endocrine markers and autoantibodies, including anti-thyroglobulin antibody, thyroid-stimulating hormone (TSH), free thyroxine (free T4), adrenocorticotropic hormone (ACTH), and cortisol.

### 2.6. Statistical Analysis

Continuous variables were expressed as the median and interquartile range [IQR] and compared using the Mann–Whitney U test. Categorical variables were compared using Fisher’s exact test. The predictive performance of baseline KL-6 was assessed using receiver operating characteristic (ROC) curve analysis, and the optimal cutoff value was determined using the Youden index. To identify independent risk factors, multiple logistic regression analysis was performed using four models. Model 1 was adjusted for age, sex, and hypertension. Model 2 was adjusted for age, sex, and lung adenocarcinoma. Model 3 evaluated the association between elevated KL-6 and irAE-LI after adjustment for age and sex. Model 4 included age, sex, lung adenocarcinoma, and elevated KL-6 to assess whether KL-6 was an independent predictor. Furthermore, to evaluate the robustness of the KL-6 signal and address potential confounding according to primary malignancy histology, a sensitivity analysis was performed. This analysis utilized a parsimonious logistic regression model including age, sex, and KL-6 in a subgroup of 75 patients, after excluding those with lung adenocarcinoma. A two-sided *p*-value of <0.05 was considered statistically significant. All analyses were performed using SPSS version 29.0 (IBM Corp., Armonk, NY, USA).

## 3. Results

### 3.1. Patient Demographics and Baseline Characteristics (Table 1)

Between November 2018 and August 2025, 81 patients with advanced malignancies who received Nivo-Ipi combination therapy at Gunma University Hospital were evaluated. The baseline clinical characteristics of the entire cohort are summarized in Table 1. The median observation period for the entire cohort was 374 days [IQR, 221–1050 days]. The median age of the patients was 67.4 years [IQR, 56.5–72.5], and the majority were male (64 patients, 79.0%). The median BMI was 21.8 kg/m^2^ [IQR, 20.1–24.7]. Regarding lifestyle factors, 55 patients (67.9%) had a history of smoking, whereas 27 patients (33.3%) had a history of alcohol use.

**Table 1:** Baseline characteristics of all patients receiving nivolumab and ipilimumab (*n* = 81).

Age, Years	67.4	[56.5–72.5]
Sex: Male, *n*	64	(79.0)
BMI, kg/m^2^	21.8	[20.1–24.7]
Observation period, days	374	[221–1050]
Primary Malignancy, *n*		
Malignant Melanoma	31	(38.3)
Lung Adenocarcinoma	6	(7.4)
Esophageal Cancer	18	(22.2)
Renal Cell Carcinoma	23	(28.4)
Others ^a^	3	(3.7)
Comorbidities, *n*		
Hypertension	51	(63.0)
Diabetes Mellitus	21	(25.9)
Dyslipidemia	15	(18.5)
Chronic Liver Disease	17	(21.0)
Lifestyle Factors, *n*		
Alcohol History	27	(33.3)
Smoking History	55	(67.9)
Occurrence of irAEs, *n*		
Any grade irAEs	45	(55.6)
irAEs requiring corticosteroids ^b^	24	(29.6)
Liver Injury (irAE-LI)	11	(13.6)
Pneumonitis	7	(8.6)
Colitis	5	(6.2)
Others ^c^	7	(8.6)

Data are expressed as number (percentage) or median [interquartile range], unless otherwise indicated. Abbreviations: BMI, body mass index; irAE, immune-related adverse event; irAE-LI, immune-related liver injury. ^a^, Others include malignant pleural mesothelioma and colorectal cancer. ^b^, A total of 30 irAE events occurred in 24 patients. ^c^, Others include myasthenia gravis, gastritis, etc. Source: Compiled by the authors.

The most frequent primary malignancy was malignant melanoma (31 patients, 38.3%), followed by renal cell carcinoma (23 patients, 28.4%), esophageal cancer (18 patients, 22.2%), and lung adenocarcinoma (6 patients, 7.4%). Other malignancies, including malignant pleural mesothelioma and colorectal cancer, accounted for three patients (3.7%). Baseline comorbidities were observed as follows: hypertension was the most common (51 patients, 63.0%), followed by diabetes mellitus (21 patients, 25.9%), chronic liver disease (17 patients, 21.0%), and dyslipidemia (15 patients, 18.5%).

irAEs of any grade were observed in 45 patients (55.6%). Notably, 24 patients (29.6%) experienced irAEs requiring systemic corticosteroid therapy, with a total of 30 distinct irAE events occurring among these individuals. Among irAE events requiring corticosteroid therapy, irAE-LI was the most frequent, accounting for 11 events (13.6%), followed by pneumonitis in seven events (8.6%), colitis in five events (6.2%), and other events, including myasthenia gravis, gastritis, and other events, in seven events (8.6%). These findings indicate that liver injury was the most frequent irAE requiring corticosteroid therapy among patients treated with Nivo-Ipi combination therapy in this study.

### 3.2. Comparison of Baseline Characteristics and Laboratory Data (Table 2)

There were no statistically significant differences between the two groups in median age (71.0 years [IQR, 66.0–78.3] in the irAE-LI group vs. 66.8 years [IQR, 59.8–72.6] in the non-irAE-LI group, *p* = 0.836) or the proportion of male patients (10 patients [90.9%] vs. 54 patients [77.1%], *p* = 0.443). The median observation period was 631 days [IQR, 195–990 days] in the irAE-LI group and 371 days [IQR, 225–1049 days] in the non-irAE-LI group, with no statistically significant difference between the groups (*p* = 0.978). The median BMI was slightly higher in the irAE-LI group, although the difference was not statistically significant (24.1 kg/m^2^ [IQR, 20.9–25.1] vs. 21.6 kg/m^2^ [IQR, 19.7–24.4], *p* = 0.168). Regarding lifestyle factors, a history of alcohol use was less frequent in the irAE-LI group (1 patient [9.1%] vs. 26 patients [37.1%], *p* = 0.089), and no significant difference was found in smoking history (9 patients [81.8%] vs. 46 patients [65.7%], *p* = 0.528).

**Table 2:** Comparison of baseline patient characteristics and laboratory data between patients with and without severe irAE liver injury (irAE-LI).

Characteristic	irAE-LI Group (*n* = 11)		Non-irAE-LI Group (*n* = 70)		*p*-Value
Demographics & Lifestyle					
Age, years	71.0	[66.0–78.3]	66.8	[59.8–72.6]	0.836
Sex: Male, *n*	10	(90.9)	54	(77.1)	0.443
BMI, kg/m^2^	24.1	[20.9–25.1]	21.6	[19.7–24.4]	0.168
Alcohol History, *n*	1	(9.1)	26	(37.1)	0.089
Smoking History, *n*	9	(81.8)	46	(65.7)	0.528
Observation period, days	631	[195–990]	371	[225–1049]	0.978
Primary Malignancy, *n*					
Malignant Melanoma	5	(45.5)	26	(37.1)	0.738
Lung Adenocarcinoma	3	(27.3)	3	(4.3)	0.030 *
Renal Cell Carcinoma	2	(18.2)	21	(30.0)	0.479
Esophageal Cancer	1	(9.1)	17	(24.3)	0.448
Comorbidities, *n*					
Hypertension	10	(90.9)	41	(58.6)	0.047 *
Diabetes Mellitus	3	(27.3)	18	(25.7)	1.000
Dyslipidemia	2	(18.2)	13	(18.6)	1.000
Chronic Liver Disease	1	(9.1)	16	(22.9)	0.443
Baseline Laboratory Data					
AST (U/L)	17	[17–20]	21	[17–27]	0.171
ALT (U/L)	19	[15–28]	19	[12–26]	0.279
ALP (U/L)	90	[83–104]	87	[72–102]	0.416
*γ*-GTP (U/L)	41	[23–58]	16	[13–20]	0.777
T-Bil (mg/dL)	0.6	[0.5–0.8]	0.5	[0.4–0.7]	0.311
Alb (g/dL)	4.0	[3.7–4.1]	3.8	[3.5–4.1]	0.391
PT-INR	0.98	[0.96–1.01]	1.00	[0.97–1.05]	0.397
CRP (mg/dL)	0.18	[0.07–0.36]	0.38	[0.06–2.51]	0.331
Biological & Immunological Markers					
KL-6 (U/mL)	419	[226–483]	216	[172–288]	0.007 *
NLR	2.76	[2.15–5.43]	3.22	[2.16–4.51]	0.335
ANA positive (≥40), *n*	1	(9.1)	7	(10.3)	1.000
Anti-Tg Ab positive, *n*	1	(16.7)	3	(5.5)	0.346
TSH (μU/mL)	1.99	[1.21–2.40]	1.51	[1.07–2.23]	0.486
Free T4 (ng/dL)	1.08	[0.96–1.20]	1.13	[0.99–1.25]	0.267
ACTH (pg/mL)	27.9	[23.1–39.8]	23.3	[15.7–38.9]	0.220
Cortisol (μg/dL)	9.9	[7.3–12.3]	10.9	[8.2–12.7]	0.763

Data are expressed as number (percentage) or median [interquartile range], unless otherwise indicated. Abbreviations: ACTH, adrenocorticotropic hormone; Alb, albumin; ALP, alkaline phosphatase; ALT, alanine aminotransferase; ANA, antinuclear antibody; Anti-Tg Ab, anti-thyroglobulin antibody; AST, aspartate aminotransferase; BMI, body mass index; Cortisol, cortisol; CRP, C-reactive protein; Free T4, free thyroxine; γ-GTP, gamma-glutamyltransferase; irAE-LI, immune-related liver injury; KL-6, Krebs von den Lungen-6; NLR, neutrophil-to-lymphocyte ratio; PT-INR, prothrombin time-international normalized ratio; T-Bil, total bilirubin; TSH, thyroid-stimulating hormone. * *p* < 0.05 indicates statistical significance based on Fisher’s exact test or Mann–Whitney U test. Source: Compiled by the authors.

Significant differences were observed in specific baseline characteristics. The prevalence of lung adenocarcinoma was significantly higher in the irAE-LI group than in the non-irAE-LI group (3 patients [27.3%] vs. 3 patients [4.3%], *p* = 0.030). Among comorbidities, hypertension was significantly more prevalent in patients who developed severe liver injury (10 patients [90.9%] vs. 41 patients [58.6%], *p* = 0.047). No significant differences were observed in other primary malignancies or comorbidities, including diabetes mellitus (3 patients [27.3%] vs. 18 patients [25.7%], *p* = 1.000), dyslipidemia (2 patients [18.2%] vs. 13 patients [18.6%], *p* = 1.000), or chronic liver disease (1 patient [9.1%] vs. 16 patients [22.9%], *p* = 0.443).

Standard biochemical markers before treatment, including AST, ALT, ALP, γ-GTP, T-Bil, Alb, and PT-INR, were comparable between the two groups. Median CRP levels were 0.18 mg/dL [IQR, 0.07–0.36] in the irAE-LI group and 0.38 mg/dL [IQR, 0.06–2.51] in the non-irAE-LI group, showing no statistically significant difference (*p* = 0.331).

Among biological and immunological markers, baseline serum KL-6 levels were significantly elevated in the irAE-LI group (median, 419 U/mL [IQR, 226–483]) compared with the non-irAE-LI group (median, 216 U/mL [IQR, 172–288]; *p* = 0.007). In contrast, the NLR did not differ significantly between the groups (2.76 [IQR, 2.15–5.43] vs. 3.22 [IQR, 2.16–4.51], *p* = 0.335).

Immunological markers, including ANA positivity (1 patient [9.1%] vs. 7 patients [10.3%], *p* = 1.000) and anti-thyroglobulin antibody positivity (1 patient [16.7%] vs. 3 patients [5.5%], *p* = 0.346), showed no significant association with the development of severe liver injury. Furthermore, endocrine markers, including TSH (1.99 μU/mL [IQR, 1.21–2.40] vs. 1.51 μU/mL [IQR, 1.07–2.23], *p* = 0.486), free T4 (1.08 ng/dL [IQR, 0.96–1.20] vs. 1.13 ng/dL [IQR, 0.99–1.25], *p* = 0.267), ACTH (27.9 pg/mL [IQR, 23.1–39.8] vs. 23.3 pg/mL [IQR, 15.7–38.9], *p* = 0.220), and cortisol (9.9 μg/dL [IQR, 7.3–12.3] vs. 10.85 μg/dL [IQR, 8.2–12.7], *p* = 0.763), were comparable between the two groups before treatment initiation.

### 3.3. Clinical Course and Treatment Response of Severe irAE-LI (Table 3)

In the irAE-LI group (*n* = 11), the median time from the initiation of Nivo-Ipi therapy to the onset of liver injury was 38 days [interquartile range (IQR), 19–67]. The median number of ICI doses received before onset was 1 [IQR, 1–2]. At the time of corticosteroid initiation, most patients had grade 3 liver injury (8 patients, 72.7%), followed by grade 4 liver injury (2 patients, 18.2%), according to CTCAE version 5.0. One patient (9.1%) began corticosteroid treatment at grade 2 because of rapid liver enzyme elevation and concurrent irAEs. The median peak ALT level during the clinical course was 393 U/L [IQR, 296–776]. Notably, no patients in the severe irAE-LI group underwent liver biopsy (0/11); the diagnosis was established by rigorously excluding other potential etiologies, including viral, obstructive, and metastatic causes, as well as the observed clinical response to treatment.

**Table 3:** Clinical course and outcomes of patients with severe irAE liver injury (*n* = 11).

Clinical Timeline		
Time to onset, days	38	[19–67]
Number of ICI doses received before onset	1	[1–2]
Onset after the first ICI dose	6	(54.5)
Severity and Pattern		
Peak ALT level, U/L	393	[296–776]
Grade at corticosteroid initiation		
Grade 2 ^a^	1	(9.1)
Grade 3	8	(72.7)
Grade 4	2	(18.2)
Pattern of liver injury		
Hepatocellular	7	(63.6)
Cholestatic	2	(18.2)
Mixed	2	(18.2)
Concurrent irAEs involving other organ systems	4	(36.4)
Pneumonitis	3	
Myositis	1	
Colitis	1	
Gastritis	1	
Initial Treatment		
Oral prednisolone, 0.5–1.0 mg/kg	8	(72.8)
Methylprednisolone, 2.0 mg/kg	2	(18.2)
Methylprednisolone pulse therapy, 1.0 g	1	(9.1)
Response and Secondary Therapy		
Steroid refractoriness or relapse	6	(54.5)
Additional immunosuppressants, MMF	3	(27.3)
Corticosteroid dose escalation, PSL/mPSL	3	(27.3)
Secondary mPSL pulse therapy	2	(18.2)
Complications and Outcomes		
CMV-DNA testing performed	8	(72.7)
CMV-DNA reactivation among tested patients ᵇ	4/8	(50.0)
Prednisolone-equivalent dose at CMV reactivation	≥40 mg/day	
Time from corticosteroid initiation to CMV reactivation, days	38–66	
Antiviral therapy with ganciclovir among CMV-positive patients	4/4	(100)
Resolution of liver injury, Grade 0–1	9	(81.8)
Death with unresolved irAE- Liver Injury	2	(18.2)

Data are expressed as number (percentage) or median [interquartile range], unless otherwise indicated. Abbreviations: ALT, alanine aminotransferase; CMV, cytomegalovirus; CTCAE, Common Terminology Criteria for Adverse Events; ICI, immune checkpoint inhibitor; irAE, immune-related adverse event; irAE-LI, immune-related liver injury; MMF, mycophenolate mofetil; mPSL, methylprednisolone; PCR, polymerase chain reaction; PSL, prednisolone. ^a^ One patient started corticosteroid treatment at Grade 2 because of rapid liver enzyme elevation or concurrent irAEs. ᵇ CMV-DNA reactivation was confirmed by polymerase chain reaction in 4 of the 8 patients tested. Source: Compiled by the authors.

The predominant biochemical pattern of liver injury was hepatocellular, observed in 7 patients (62.6%), whereas cholestatic and mixed patterns were each observed in 2 patients (18.2%). Furthermore, according to the detailed clinical review, concurrent irAEs involving other organ systems were identified in 4 patients (36.4%); these included pneumonitis in 3 patients, as well as myositis, colitis, and gastritis.

All 11 patients received systemic corticosteroid therapy as the initial intervention. The initial corticosteroid regimens consisted of oral prednisolone (0.5–1.0 mg/kg) in 8 patients (72.7%), intravenous methylprednisolone (mPSL; 2.0 mg/kg) in 2 patients (18.2%), and high-dose mPSL pulse therapy (1.0 g) in 1 patient (9.1%). This use of initial pulse therapy was limited to the early study period (before 2022); since 2023, our institution has standardized the starting dose to 1.0 mg/kg without routine pulse therapy, in accordance with updated Japanese and international guidelines.

A notable observation in this cohort was the high frequency of steroid-refractory disease or biochemical relapse during tapering, which occurred in 6 of 11 patients (54.5%). This rate is higher than those typically reported for high-grade ICI-induced hepatotoxicity in general clinical series. To manage these refractory or relapsing cases, secondary therapies were required, including the addition of MMF in 3 of the 6 patients (50.0%), corticosteroid dose escalation in 3 of the 6 patients (50.0%), and secondary mPSL pulse therapy in 2 of the 6 patients (33.3%).

Regarding complications during intensive immunosuppression, CMV reactivation was evaluated in 8 of 11 patients (72.7%). Testing was not performed systematically in all cases but was prompted by clinical indicators such as a pseudo-refractory course despite high-dose steroids, or the development of symptomatic cytopenia or gastrointestinal symptoms. CMV reactivation, confirmed by pp65 antigenemia or quantitative PCR, was detected in 4 of 8 tested patients (50.0%). All four CMV-positive patients were receiving a PSL-equivalent dose of ≥40 mg/day at the time of reactivation and subsequently received antiviral therapy with ganciclovir. Clinical improvement of liver injury, defined as resolution to Grade 0–1, was achieved in 9 of 11 patients (81.8%). The remaining two patients died with unresolved irAE-LI because of cancer progression or infectious complications.

### 3.4. Risk Factor Analysis for Severe irAE Liver Injury (Figure 2 and Table 4 and Table 5)

**Figure 2:**
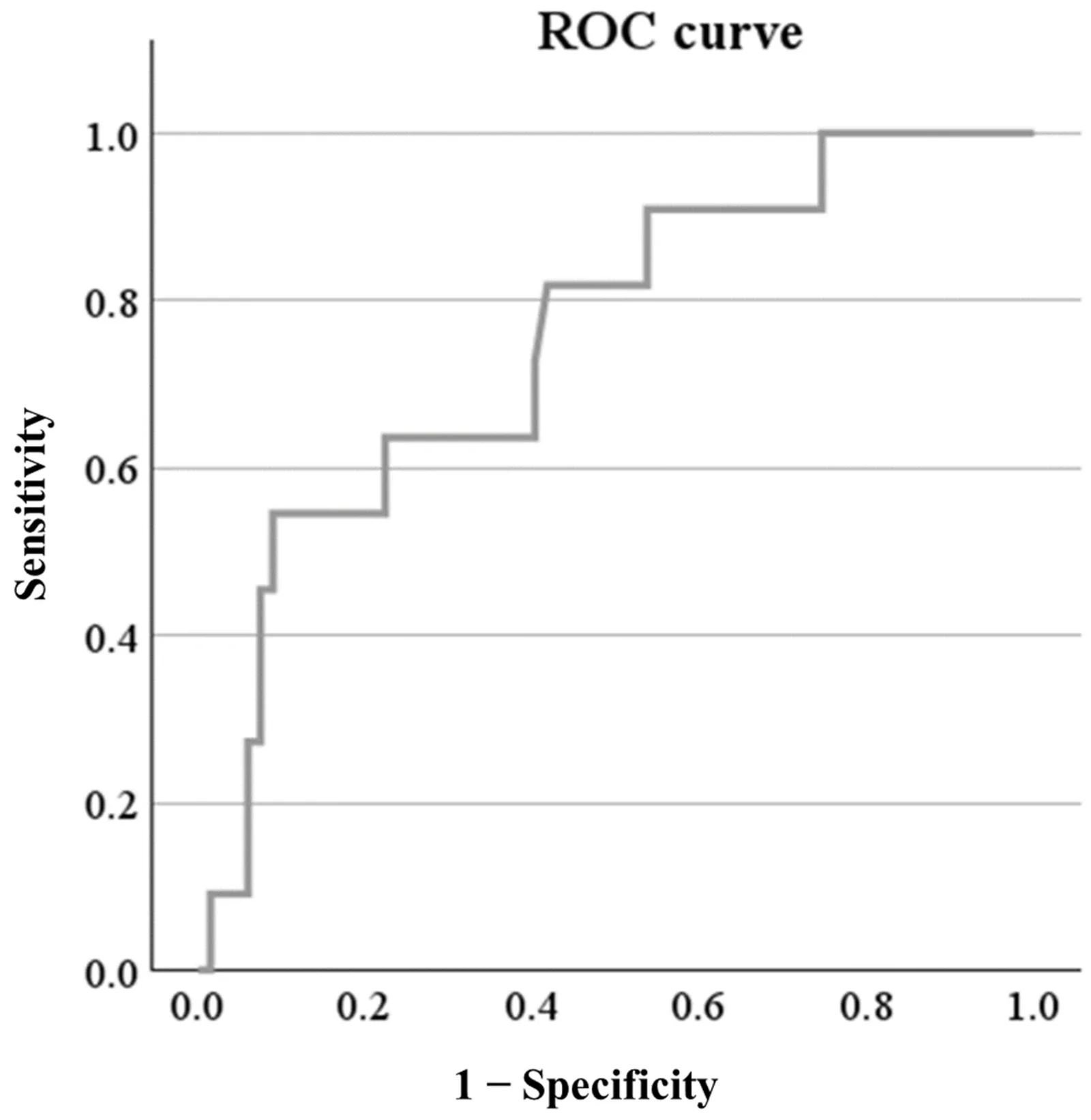
Receiver operating characteristic (ROC) curve of baseline serum KL-6 levels for predicting severe immune-related adverse event liver injury (irAE-LI).

To evaluate the predictive performance of baseline serum KL-6 for severe irAE-LI, ROC curve analysis was conducted (Figure 2). The area under the curve (AUC) was 0.755 (95% CI, 0.575–0.935), indicating moderate-to-high discriminatory ability. The optimal cutoff value determined by the Youden index was 418 U/mL, yielding a sensitivity of 0.545 and a specificity of 0.910. For greater clinical applicability in screening high-risk patients, a threshold of 400 U/mL was adopted for subsequent multivariable analyses.

The area under the curve (AUC) was 0.755 (95% confidence interval [CI]: 0.575–0.935). At the optimal cut-off value of 418 U/mL (determined by the Youden index), the sensitivity was 0.545 and the specificity was 0.910 (1 − specificity = 0.090). These data indicate that baseline serum KL-6 is a significant predictor for the development of severe hepatotoxicity requiring corticosteroid therapy following nivolumab and ipilimumab combination treatment. Source: Compiled by the authors.

This exploratory multivariable analysis was performed using logistic regression models to identify potential preliminary risk signals for severe irAE-LI (Table 4).

**Table 4:** Multivariate logistic regression analysis of risk factors for severe irAE liver injury.

Variable	Model 1	Model 2	Model 3	Model 4
Age	0.96 (0.91–1.02) [0.230]	0.98 (0.93–1.04) [0.549]	0.96 (0.90–1.02) [0.205]	0.96 (0.90–1.03) [0.226]
Sex (Male)	2.23 (0.25–19.87) [0.473]	2.28 (0.26–20.08) [0.458]	3.66 (0.37–35.89) [0.266]	2.83 (0.28–28.36) [0.377]
Hypertension	8.71 (0.95–80.12) [0.056]	—	—	—
Lung Adenocarcinoma	—	7.35 (1.22–44.22) [0.029 *]	—	3.56 (0.45–28.30) [0.231]
KL-6 ≥ 400 U/mL	—	—	16.18 (3.04–86.17) [0.001 *]	12.60 (2.32–68.60) [0.003 *]

Values represent Odds Ratio (95% Confidence Interval) [*p*-value]. Abbreviations: CI, confidence interval; KL-6, Krebs von den Lungen-6; OR, odds ratio. * *p* < 0.05 indicates statistical significance. Source: Compiled by the authors.

- Models 1 and 2: Clinical factors. After adjustment for age and sex, hypertension in Model 1 showed a trend toward an increased risk but did not reach statistical significance (OR, 8.71; *p* = 0.056). In Model 2, lung adenocarcinoma was significantly associated with severe irAE-LI (OR, 7.35; *p* = 0.029).
- Model 3: Impact of KL-6. After adjustment for age and sex, baseline KL-6 ≥ 400 U/mL was significantly associated with severe irAE-LI, with an odds ratio of 16.18 (*p* = 0.001).
- Model 4: Final integrated model. In the final model, which included age, sex, lung adenocarcinoma, and baseline KL-6 levels of ≥400 U/mL, baseline KL-6 levels of ≥400 U/mL showed a strong preliminary signal for severe irAE-LI (OR, 12.60; 95% CI, 2.32–68.60; *p* = 0.003), whereas lung adenocarcinoma was no longer statistically significant (*p* = 0.231).

To address the risk of statistical overfitting due to the small number of events (*n* = 11) and to minimize potential confounding by primary malignancy, we conducted a sensitivity analysis excluding the six patients with lung adenocarcinoma. In this subgroup (*n* = 75, events = 8), a more parsimonious multivariable model adjusted for age and sex showed that baseline KL-6 levels of ≥400 U/mL remained highly and significantly associated with severe irAE-LI (OR, 13.21; 95% CI, 2.21–79.16; *p* = 0.005), as shown in Table 5.

**Table 5:** Multivariate logistic regression analysis of risk factors for severe irAE liver injury excluding patients with lung adenocarcinoma (*n* = 75).

Variable	
Age	0.97 (0.97–1.04) [0.442]
Sex (Male)	2.98 (0.29–30.94) [0.360]
KL-6 ≥ 400 U/mL	13.21 (2.21–79.16) [0.005 *]

Values represent Odds Ratio (95% Confidence Interval) [*p*-value]. Abbreviations: CI, confidence interval; KL-6, Krebs von den Lungen-6; OR, odds ratio. * *p* < 0.05 indicates statistical significance. Source: Compiled by the authors.

We re-evaluated baseline host factors related to metabolic health and underlying liver status. Approximately 20.0% of the entire cohort (17/81) had chronic liver disease (CLD), primarily fatty liver diseases, including metabolic dysfunction-associated steatotic liver disease (MASLD) and Met-ALD. However, baseline CLD was not significantly associated with the development of severe irAE-LI (9.1% in the irAE-LI group vs. 22.9% in the non-irAE-LI group; *p* = 0.443; Table 2). Among the individual metabolic components, only hypertension was significantly associated with severe irAE-LI.

## 4. Discussion

The key finding of this study was that baseline serum KL-6 levels were identified as a potential biomarker associated with a strong preliminary risk signal for severe irAE-LI and may be useful for risk stratification before the initiation of Nivo-Ipi combination therapy. Although liver injury following PD-1 or PD-L1 inhibitor monotherapy typically develops 6–14 weeks after treatment initiation, dual immune checkpoint blockade with Nivo-Ipi has been reported to increase the risk of liver injury and alter its temporal profile, leading to earlier onset and greater clinical severity [6]. In the present cohort, the median time to onset in the irAE-LI group was 38 days, and 6 of 11 patients (54.5%) developed severe liver injury after the first dose of Nivo-Ipi. These findings are consistent with previous reports indicating that combination therapy can induce liver toxicity as early as 3–9 weeks after treatment initiation [5,6,18,19].

The incidence and severity of hepatotoxicity are also higher with dual immune checkpoint blockade. Clinical studies have reported that the frequency of liver injury of any grade increases from 1 to 15% with monotherapy to 3–30% with combination therapy [9]. Furthermore, the incidence of grade 3 or 4 hepatotoxicity has been estimated to be approximately 13–30% in patients receiving Nivo-Ipi, compared with <10% in those receiving single-agent therapy [8]. Major clinical trials across various cancer types have also underscored this risk. For example, the CheckMate 067 trial in patients with melanoma reported grade 3–4 treatment-related adverse events in 59% of the combination-therapy group, including liver-related deaths [1]. Similarly, the CheckMate 214 trial in patients with renal cell carcinoma [2], the CheckMate 227 trial in patients with non-small cell lung cancer [20], and the CheckMate 9DW trial in patients with hepatocellular carcinoma [21] documented severe or fatal hepatic events, including immune-mediated hepatitis and liver failure. These data highlight the potentially life-threatening nature of these toxicities in high-risk populations and indicate that clinicians should perform intensive liver enzyme monitoring from the first cycle of Nivo-Ipi to facilitate early diagnosis and the prompt initiation of high-dose corticosteroids when required to manage rapidly progressive or severe hepatotoxicity [6].

While KL-6 has historically been a biomarker centered in Asia, its external applicability is increasing. Recent global reviews, such as those by Fadelelmoula et al. [22], highlight that standardized KL-6 assays are now increasingly available in parts of Europe and the Asia-Pacific region, demonstrating high diagnostic accuracy (>80%) for interstitial lung diseases across different clinical settings. This suggests that the clinical translation of our findings outside Japan is becoming more feasible.

KL-6 is a sialylated glycoprotein primarily used as a sensitive biomarker for ILD and ICI-induced pneumonitis, reflecting injury to alveolar type II epithelial cells [14,15]. In the context of liver injury, the predictive value of KL-6 is noteworthy because standard biochemical markers, including baseline transaminases (AST and ALT), biliary enzymes (ALP and γ-GTP), and total bilirubin, showed no significant association with the subsequent development of severe irAE-LI in this cohort. This finding is consistent with previous real-world data indicating that baseline hepatic status does not necessarily correlate with the risk of immune-mediated liver injury [5,6,23].

One major concern was whether KL-6 independently predicts hepatotoxicity or simply acts as a surrogate marker for underlying lung adenocarcinoma or interstitial lung disease (ILD). However, our findings argue against this “surrogate marker” hypothesis. In the final multivariable model (Model 4), lung adenocarcinoma was no longer statistically significant after baseline KL-6 levels were included (*p* = 0.231). More importantly, our sensitivity analysis excluding the 6 patients with lung adenocarcinoma (Table 5) showed that baseline KL-6 levels of ≥400 U/mL remained highly and significantly associated with severe liver injury (Odds Ratio 13.21, 95% CI 2.21–79.16, *p* = 0.005). This consistency suggests that elevated KL-6 may capture a specific biological risk signal for multi-organ inflammation—potentially reflecting a systemically primed immune state—rather than being limited to pulmonary pathology.

Given that Nivo-Ipi combination therapy carries a high incidence of Grade 3–4 hepatotoxicity (up to 30%) [4,5,9] and often results in rapid, severe onset after only a single dose [5,6], the ability to risk-stratify patients before treatment is of paramount clinical importance. Identifying patients with baseline KL-6 levels of ≥400 U/mL, which showed a potential biomarker demonstrating a strong preliminary risk signal, allows clinicians to implement more intensive monitoring of liver function and adopt a lower threshold for initiating immunosuppressive therapy, potentially improving the safety profile of dual checkpoint blockade [6,14,15]. Although KL-6 has historically been utilized primarily in Asia, recent evidence indicates that standardized assays are increasingly available in Europe and the Asia-Pacific region, supporting the potential international applicability of our findings.

A significant observation in this study was the high frequency of steroid-refractory disease or relapse during corticosteroid tapering, which occurred in 6 of 11 patients (54.5%) with severe irAE-LI. Notably, although these patients had received high-dose corticosteroids (PSL 1 mg/kg or mPSL 2 mg/kg) in accordance with established clinical practice guidelines [6,18,19], this rate was higher than the 20–30% reported in general clinical series of high-grade ICI-induced hepatotoxicity [4,5]. This disparity may partly reflect the clinical intractability of liver injury triggered by the synergistic immune activation associated with dual checkpoint blockade using Nivo-Ipi [3,5]. CMV reactivation may complicate the clinical course. Although most cases of irAE-LI resolve with initial corticosteroid therapy [6,9], combination regimens are associated with more aggressive inflammatory profiles and a higher incidence of grade 3–4 events compared with monotherapy [1,4,5,9].

Although we acknowledge that the 7-year inclusion period (2018–2025) introduced potential historical confounding bias due to evolving treatment protocols, the management of these steroid-refractory or relapsing cases in our cohort was generally consistent with established clinical practice guidelines regarding initial treatment [6,18,19]. Specifically, our strategy for severe irAE-LI evolved from the occasional use of initial or secondary mPSL pulse therapy until 2022 to a more standardized approach centered on PSL at 1.0 mg/kg without routine pulse therapy from 2023 onward. This transition was a strategic shift toward alignment with the latest expert consensus—including the Japanese Society of Clinical Oncology [18] and the Japan Society of Hepatology guidelines [24], which emphasize avoiding excessive immunosuppression when the benefits of pulse therapy for irAE-LI remain unproven. Because the addition of MMF, which is recommended when the initial response to corticosteroids is inadequate, is off-label in Japan, our early study period included cases in which corticosteroid dose escalation or mPSL pulse therapy was selected instead. In the present study, among patients requiring second-line intervention for steroid-refractory disease or relapse, 3 of the 6 patients (50.0%) received MMF, whereas the remaining 3 patients were managed without MMF, using corticosteroid dose escalation and/or mPSL pulse therapy. Clinical improvement of liver injury was observed in 9 of the 11 patients (81.8%), whereas the remaining two patients died before resolution of liver injury.

MMF is currently a preferred second-line agent in many guidelines [3,4,5] because it inhibits lymphocyte proliferation and targets the T-cell-mediated pathophysiology of irAE-LI [1,3,5]. Given the high frequency of steroid-refractory disease observed in this Nivo-Ipi cohort (54.5%), our institution has shifted its clinical practice toward prioritizing early MMF addition in patients who exhibit an inadequate initial response or biochemical relapse during tapering.

The management of refractory irAE-LI is further complicated by the risks associated with prolonged high-dose corticosteroid exposure [4,6,19]. Recent retrospective data suggest that initial corticosteroid doses exceeding 1.5 mg/kg do not lead to more rapid normalization of liver enzyme levels but may significantly increase the risk of corticosteroid-related complications, such as hyperglycemia and serious infections [25]. In the present study, the frequent steroid-refractory disease or relapse observed after corticosteroid initiation or during tapering suggests that, although high-dose corticosteroids may be necessary to control initial inflammation, the subsequent tapering phase should be conducted cautiously with individualized monitoring to account for potential biochemical flares [6,19,26,27]. In line with these global trends and the 2025 guidelines of the Japan Society of Hepatology [24], our institution has now standardized its clinical practice by prioritizing PSL at 1.0 mg/kg as first-line therapy for grade ≥ 3 severe irAE-LI, with careful monitoring for secondary complications such as CMV reactivation.

Finally, the clinical picture of “steroid refractoriness” may be complicated by secondary factors, most notably CMV reactivation [6,10,20]. In the present study, CMV reactivation—diagnosed using an integrated clinical and microbiological approach (pp65 antigenemia, ≥1 positive cell/150,000 WBCs, or quantitative real-time PCR, ≥2.0 log_10_ copies/mL)—was identified in 4 of 8 tested patients (50.0%) who were receiving high-dose corticosteroids. Notably, CMV testing was not performed systematically but was prompted by clinical suspicion, including cases showing a “pseudo-refractory” course despite intensified corticosteroid therapy, or the development of symptomatic cytopenia or gastrointestinal symptoms. CMV infection can clinically mimic worsening irAE-LI, leading to a “pseudo-refractory” state in which liver enzyme levels fail to improve or even deteriorate despite intensified immunosuppression [10,19]. These findings suggest that, in patients with Nivo-Ipi-induced liver injury undergoing immunosuppressive therapy, clinicians should prioritize excluding CMV reactivation using PCR or antigenemia assays when liver injury deteriorates before further escalation of immunosuppression. This is because clinical resolution may require antiviral therapy with agents such as ganciclovir rather than corticosteroid dose escalation or the addition of other immunosuppressants [5,6,18,19].

In our univariable analysis, both lung adenocarcinoma and hypertension were identified as baseline clinical factors significantly associated with the development of severe irAE-LI. The association between lung adenocarcinoma and liver toxicity is noteworthy. Although previous studies have suggested that certain primary malignancies may confer an increased risk of organ-specific irAEs, such as pneumonitis in patients with lung cancer [5,6], a specific link to immune-mediated hepatotoxicity remains less well characterized [5,13].

Regarding hypertension, our study found that it was significantly more prevalent in the irAE-LI group than in the non-irAE-LI group (Table 2). Although hypertension showed only a trend toward an increased risk in Model 1 of the multivariable analysis (OR, 8.71; *p* = 0.056), its high frequency (90.9%) among patients who developed severe liver injury warrants clinical attention. Emerging evidence, including the large-scale analysis by Li et al. [28], which identified hypertension as an independent risk factor for irAEs across multiple organs, and the study by Lei et al. [29] on metabolic syndrome, suggests that metabolic comorbidities may predispose patients to more frequent immune-mediated events. Furthermore, we re-evaluated baseline host factors related to metabolic health and underlying liver status. Approximately 20.0% of the entire cohort had chronic liver disease (CLD), primarily fatty liver diseases, including metabolic dysfunction-associated steatotic liver disease (MASLD) and Met-ALD. Although a report by Sawada et al. suggested that NAFLD is a potential risk factor for liver injury specifically during anti-PD-1 monotherapy [12], we found no significant association between baseline CLD and the development of severe irAE-LI in this cohort receiving dual checkpoint blockade (9.1% vs. 22.9%, *p* = 0.443). This may imply that the intense immune activation triggered by Nivo-Ipi could outweigh the metabolic predisposition observed in monotherapy settings. The association between hypertension and irAE-LI may reflect underlying vascular endothelial vulnerability or chronic low-grade inflammation, which could exacerbate tissue-damaging immune responses triggered by Nivo-Ipi combination therapy [7]. These findings suggest that although primary malignancy and common comorbidities such as hypertension may provide initial clues for risk stratification, integrating biological markers such as KL-6 may enable more precise identification of patients at high risk of severe hepatotoxicity following dual checkpoint blockade [6].

This study has several limitations that should be acknowledged. First, the total number of events was small (*n* = 11), resulting in a low events-per-variable ratio in our multivariable models. Therefore, these results should be interpreted as exploratory signals rather than definitive predictive factors, and they may be affected by statistical overfitting. Although the overall cohort of 81 consecutive patients receiving Nivo-Ipi may reflect real-world practice, the small number of events may limit the statistical power and generalizability of the findings. Second, the 7-year inclusion period (2018–2025) may have introduced potential historical confounding bias, as treatment protocols evolved from the occasional use of initial mPSL pulse therapy until 2022 to a more standardized approach centered on PSL at 1.0 mg/kg in accordance with updated clinical guidelines. Third, the study population was heterogeneous and included various malignancies. Although the robustness of the baseline KL-6 signal was supported by a sensitivity analysis excluding patients with lung adenocarcinoma, further validation in larger, cancer-specific cohorts is warranted. Fourth, because this was a retrospective study, certain specialized investigations were not systematically performed. CMV testing was clinically indicated rather than systematic (performed in 8 of 11 cases); thus, the reported reactivation rate may be subject to selection bias and should be considered a preliminary signal in high-risk subgroups. Finally, liver biopsy was not performed in any of the patients with severe irAE-LI (0/11). Although the clinical decision to forgo biopsy was based on the successful exclusion of other etiologies through non-invasive means and the urgent need to initiate corticosteroids for rapidly progressive injury, this lack of histological data prevented us from characterizing the pathological patterns of liver injury across our cohort.

## 5. Conclusions

In conclusion, Nivo-Ipi combination therapy was associated with early-onset and severe immune-related liver injury, which frequently occurred after the first dose. This exploratory study identified baseline serum KL-6 levels of ≥400 U/mL as a potential biomarker associated with a strong preliminary risk signal for severe irAE-LI requiring corticosteroid therapy. Furthermore, liver injury induced by dual checkpoint blockade may be associated with a high rate of steroid-refractory disease or relapse during corticosteroid tapering, often necessitating prolonged immunosuppression, which may increase the risk of CMV reactivation and contribute to a pseudo-refractory clinical course. These findings suggest that pretreatment serum KL-6 screening, intensive monitoring of liver enzyme levels from the initiation of therapy, and early consideration of mycophenolate mofetil and CMV screening may be important strategies for the safe management of patients undergoing dual immune checkpoint inhibition. However, given the statistical limitations of this study, these preliminary findings warrant rigorous validation in larger, prospective multicenter cohorts.

## Data Availability

The data presented in this study are available on request from the corresponding author due to ethical and privacy restrictions (to protect patient confidentiality in accordance with the institutional ethics committee approval).

## Abbreviations

The following abbreviations are used in this manuscript:

ACTH	adrenocorticotropic hormone
AIH	autoimmune hepatitis
Alb	albumin
ALP	alkaline phosphatase
ALT	alanine aminotransferase
ANA	antinuclear antibody
AST	aspartate aminotransferase
AUC	area under the curve
BMI	body mass index
CI	confidence interval
CMV	cytomegalovirus
CRP	C-reactive protein
CTCAE	Common Terminology Criteria for Adverse Events
DILI	drug-induced liver injury
γ-GTP	gamma-glutamyltransferase
ICI	immune checkpoint inhibitor
ILD	interstitial lung disease
IQR	interquartile range
irAE	immune-related adverse event
irAE-LI	immune-related liver injury
KL-6	Krebs von den Lungen-6
MMF	mycophenolate mofetil
mPSL	methylprednisolone
MRCP	magnetic resonance cholangiopancreatography
Nivo-Ipi	nivolumab and ipilimumab
NLR	neutrophil-to-lymphocyte ratio
OR	odds ratio
PCR	polymerase chain reaction
PD-1	programmed cell death-1
PSL	prednisolone
PT-INR	prothrombin time-international normalized ratio
ROC	receiver operating characteristic
RUCAM	Roussel Uclaf Causality Assessment Method
T-Bil	total bilirubin
TSH	thyroid-stimulating hormone
